# Cotton *KNL1*, encoding a class II KNOX transcription factor, is involved in regulation of fibre development

**DOI:** 10.1093/jxb/eru182

**Published:** 2014-05-15

**Authors:** Si-Ying Gong, Geng-Qing Huang, Xiang Sun, Li-Xia Qin, Yang Li, Li Zhou, Xue-Bao Li

**Affiliations:** Hubei Key Laboratory of Genetic Regulation and Integrative Biology, College of Life Sciences, Central China Normal University, Wuhan 430079, China

**Keywords:** Cotton (*Gossypium hirsutum*), dominant repression of gene expression, fibre development, knotted-like homeobox protein (KNOX), protein–protein interaction, secondary cell wall (SCW) biosynthesis.

## Abstract

In this study, the *GhKNL1* (*KNOTTED1-LIKE*) gene, encoding a classical class II KNOX protein was identified in cotton (*Gossypium hirsutum*). *GhKNL1* was preferentially expressed in developing fibres at the stage of secondary cell wall (SCW) biosynthesis. GhKNL1 was localized in the cell nucleus, and could interact with GhOFP4, as well as AtOFP1, AtOFP4, and AtMYB75. However, GhKNL1 lacked transcriptional activation activity. Dominant repression of *GhKNL1* affected fibre development of cotton. The expression levels of genes related to fibre elongation and SCW biosynthesis were altered in transgenic fibres of cotton. As a result, transgenic cotton plants produced aberrant, shrunken, and collapsed fibre cells. Length and cell-wall thickness of fibres of transgenic cotton plants were significantly reduced compared with the wild type. Furthermore, overexpression and dominant repression of *GhKNL1* in *Arabidopsis* resulted in a reduction in interfascicular fibre cell-wall thickening of basal stems of transgenic plants. Complementation revealed that *GhKNL1* rescued the defective phenotype of *Arabidopsis knat7* mutant in some extent. These data suggest that GhKNL1, as a transcription factor, participates in regulating fibre development of cotton.

## Introduction

The plant cell wall is a complex and dynamic structure that not only regulates cell growth and provides structural and mechanical support to the plant, but also acts as a barrier against the environment and potentially organisms (Somerville *et al.*, 2004). Cell walls at secondary growth, which widely exists in herbaceous plants and trees, are usually divided into two categories: primary cell wall (PCW) and secondary cell wall (SCW). After cessation of cell growth, SCW is deposited inside the PCW in certain cell types, such as fibres and tracheary elements.

Cotton (*Gossypium hirsutum* L.) is the most important textile fibre crop in the world. Cotton fibre, a highly elongated and thickened single cell derived from the ovule epidermis, provides an excellent system for study on cell elongation and SCW biosynthesis (Kim and Triplett, 2001; Haigler *et al.*, 2012). Fibre development includes four distinct and overlapping stages: initiation, elongation (PCW biosynthesis), SCW biosynthesis/thickening, and maturation. From 0 days post anthesis (DPA) to approximately 21 DPA, fibre cells dramatically elongate and large amounts of cell-wall components are required to be synthesized in fibre cells (Liu *et al.*, 2000; Haigler *et al.*, 2012). The PCW contains ~22% crystalline cellulose fibrils surrounded by xyloglucan and pectin (Meinert and Delmer, 1977; Singh *et al.*, 2009). Cellulose is abundantly synthesized and deposited on cell walls in an orderly manner at the SCW stage (Wilkins and Arpat, 2005). After 45 DPA, fibre cells enter a period of dehydration and maturation. In mature fibres, final dry weight can be attributed to 95% cellulose (Timpa and Triplett, 1993).

It is believed that a large number of genes are involved in regulation of fibre developmental process. In the past decade, it has been reported that some genes play important roles in early fibre development. For instance, the MYB transcription factors (TFs) GhMYB25 and GhMYB25-like regulate early fibre development (Machado *et al.*, 2009). The sucrose synthase gene (*Sus*) affects cotton fibre initiation and elongation and seed development (Ruan *et al.*, 2003). *GhXTH1*-overexpressing transgenic plants have about 2-fold-higher xyloglucan endotransglycosylase activity and 15–20% longer fibres than those of the wild type (Eklöf and Brumer, 2010; Lee *et al.*, 2010). A series of complex regulations at the PCW stage finally affect fibre cell density and length. Cellulose organizes into microfibrils in cell walls, providing strength and flexibility to plants (Haigler *et al.*, 2012). Cellulose synthesis and SCW deposition in later phases determine cotton fibre yield and quality. Until now, a few proteins have been found to be involved in the process of SCW synthesis. Members of the chitinase-like (CTL) group, CTL1 and CTL2, are expressed preferentially in cotton cells in the SCW and are essential for cellulose synthesis in the SCW (Zhang *et al.*, 2004). SusC is targeted to the fibre cell wall during SCW synthesis in cotton (Brill *et al.*, 2011). Cellulose synthase A2 (GhCesA2)-A_T_ and GhCesA2-D_T_, associated with SCW cellulose biosynthesis in developing fibres, might affect cotton fibre properties (Kim *et al.*, 2012). Although some TFs have been characterized to play roles in SCW biosynthesis during plant development, little is known in detail about how the TFs are involved in regulating SCW formation of cotton fibres. Characterizing these TFs related to SCW biosynthesis of fibre cells will enable further understanding of the molecular mechanism of fibre development and improve cotton fibre quality by genetic manipulation.

Previous studies indicated that NAC and MYB TFs participate in the complex process of transcriptional regulation of SCW biosynthesis in *Arabidopsis* and some woody plants (Zhong and Ye, 2007). Recently, a study reported that the homeodomain TF KNAT7 is involved in the regulation of SCW biosynthesis in *Arabidopsis* and is functionally conserved in *Populus* (Li *et al.*, 2012). The homeobox gene family, which usually contains a homeodomain, can be classified into 14 distinct classes according to sequence–evolutionary analysis, conserved intron–exon structure, and unique codomain architectures (Mukherjee *et al.*, 2009). Although a lot of homeobox genes have been reported from plants, a complete survey and classification of all homeobox genes in plant species from disparate evolutionary groups is lacking. KNOX (Knotted related homeobox) and BEL-like (BELL) belong to a subfamily of the THREE AMINO ACID LOOP EXTENSION (TALE) family of plant homeodomain proteins (Burglin, 1997). *KNOX* genes fall into three classes on the basis of the similarity of certain residues within the homeodomain, intron positions, and expression patterns (Kerstetter *et al.*, 1994; Reiser *et al.*, 2000; Magnani and Hake, 2008). Class I KNOXs, such as STM, BP/KNAT1 (*KN*OTTED1-LIKE in *A*RABIDOPSIS *T*HALIANA), KNAT2, and KNAT6 in *Arabidopsis*, act as transcriptional activators or repressors in meristem function, control of leaf shape, and hormone homeostasis (Hake *et al.*, 2004; Hay and Tsiantis, 2010; Qi and Zheng, 2013). Expression of class II *KNOX* genes is more widespread and the roles of these genes are much more diverse. A previous study suggested that *Arabidopsis KNAT3*, *KNAT4*, and *KNAT5* are involved in root development (Truernit *et al.*, 2006). *AtKNAT7* and *PoptrKNAT7* negatively regulate SCW formation in *Arabidopsis* and *Populus* (Li *et al.*, 2012). Li *et al.* (2011) proposed that KNAT7 forms a functional complex with Ovate Family Protein 4 (OFP4) to regulate aspects of SCW formation. Bhargava *et al.* (2013) found that KNAT7 interacts with MYB75 and modulates SCW deposition both in stems and seed coat in *Arabidopsis*. *KNATM*, a class III *KNOX* gene, plays a role in leaf proximal–distal patterning, serrations, and compound leaf development (Magnani and Hake, 2008).

The current study reports a cotton class II *KNOX* gene (designated as *GhKNL1*) that is preferentially expressed in developing fibres at the SCW stage. Further study revealed that GhKNL1 functions in regulating fibre development of cotton.

## Materials and methods

### Plant materials

Sterilized seeds of cotton (*G. hirsutum* cv. Coker 312) were germinated on half-strength Murashige and Skoog (MS) medium under a 16/8h light/dark cycle at 28 °C for 6 days. Roots, cotyledons, and hypocotyls were collected from these sterile cotton seedlings, and the other tissues (such as leaves, stems, petals, anthers, ovules, and fibres) were derived from cotton plants grown in the field under normal farming conditions.

*Arabidopsis thaliana* (Columbia ecotype) was used as in this study. *Arabidopsis* transformation was performed by the floral-dip method as described previously (Gong *et al.*, 2012). Seeds of the T-DNA insertion mutant of *Arabidopsis KNAT7* (SALK-110899, designated as *knat7-3*) were obtained from the *Arabidopsis* Biological Resource Center (ABRC, http://abrc.osu.edu/) and were identified using SIGnal database (http://signal.salk.edu/). T-DNA insertion and suppression of *KNAT7* expression in transgenic plants were confirmed using flanking gene-specific primers (Supplementary Table S1 available at *JXB* online). Homozygous lines were identified for phenotype characterization. For phenotypic analysis and RNA isolation, plants grown under a 16/8h light/dark cycle (22 °C) were sampled 2cm from the bottom of 6-week-old inflorescence stems.

### Isolation of *GhKNL1* cDNA and genomic DNA

More than 4000 cDNA clones were randomly selected from the cotton fibre cDNA library for sequencing. One cDNA clone encoding a homeobox domain protein was identified and designated as *GhKNL1*. Subsequently, the genomic DNA sequence of *GhKNL1* was amplified by PCR using gene-specific primers (Supplementary Table S1 available at *JXB* online) and cloned into pBluescript II SK (pSK) vector for sequencing.

### DNA and protein sequence analysis

DNA and protein sequences were analysed using DNASTAR software (DNAStar, MD, USA). Protein domains and significant sites were identified using Motif Scan (http://myhits.isb-sib.ch/cgi-bin/motif_scan). Sequence alignment was performed using ClustalW (http://www.ebi.ac.uk). Phylogenetic analysis was performed to determine evolutionary relationships among protein sequences. A minimum evolution tree was generated using MEGA version 1 (http://www.megasoftware.net/). A bootstrap analysis with 1000 replicates was performed to assess the statistical reliability of the tree topology.

### Subcellular localization

The coding sequences of *GhKNL1* and *GhOFP4* were inserted into modified pBI121 (pMD)-eGFP vector at a position upstream of *eGFP*. Primers are listed in Supplementary Table S1 available at *JXB* online. The constructs were introduced into cotton by the method described previously (Li *et al.*, 2002). After subculture for 2–3 months, stably transformed callus cells were selected on MS selective medium. The transformed cells were stained by 4′6-diamidino-2-phenylindole (DAPI, a nucleus-specific dye). Subsequently, GFP fluorescence and DAPI fluorescent staining in the transformed cells were examined under a SP5 Meta confocal laser microscope (Leica, Wetzlar, Germany). SP5 software (Leica) was employed to record and process the digital images taken.

### Transcriptional activation and yeast two-hybrid analysis

For transcriptional activation assay, the coding sequence of *GhKNL1* was cloned into yeast vector PGBKT7 (bait vector), and the construct was transferred into yeast strains Y187 and AH109, creating fusions to the binding domain and activation domain, respectively, of the yeast transcriptional activator GAL4. AH109 transformants were grown on double dropout medium (DDO medium; SD/–Ade/–Trp; BD Biosciences Clontech, Palo Alto, CA, USA) and Y187 transformants were further confirmed with colour change on a β-galactosidase filter paper using the flash-freezing filter assay (Zhang *et al.*, 2010).

For yeast two-hybrid assay of protein–protein interactions, coding sequences of *GhKNL1*, segmented *GhKNL1*, *GhOFP4*, *AtOFP1*, *AtOFP4*, and *AtMYB75* were cloned into pGBKT7 vector (bait vector) and pGADT7 vector (prey vector), creating fusions to the binding domain and activation domain, respectively, of the yeast transcriptional activator GAL4. pGBKT7-KNL1 constructs were introduced to strain Y187 and pGADT7-KNL1, -GhOFP4, *-*AtOFP1, -AtOFP4, and -AtMYB75 constructs were introduced to strain AH109, using the high-efficiency lithium acetate transformation procedure (Gietz *et al.*, 1992). Mating reactions were performed between the two haploid strains. Positive clones were selected on QDO medium (SD/–Trp/–Leu/–His/–Ade; BD Biosciences Clontech, www.bdbiosciences.com) at 30 °C. After further cultivation for 7 days, independent positive clones were restreaked on QDO two or three times and LacZ activity was further tested by the flash-freezing filter assay (Zhang *et al.*, 2010). Primers are listed in Supplementary Table S2 available at *JXB* online.

### Bimolecular fluorescence complementation analysis of interaction between GhKNL1 and GhOFP4

pUC-SPYNE-GhKNL1 and pUC-SPYCE-GhOFP4 vectors were constructed using gene-specific primers (Supplementary Table S2 available at *JXB* online). The constructs were then introduced and transiently coexpressed in onion epidermal cells by DNA particle bombardment according to the manufacturer’s instructions (Biolistic PDS-1000/He Particle Delivery System, Bio-Rad, USA). YFP fluorescence in the transformed cells was detected under confocal laser scanning microscopy using bZIP63 dimerization as a positive control and pUC-SPYNE-GhKNL1+pUC-SPYCE and pUC-SPYNE+pUC-SPYCE-GhOFP4 as negative controls.

### Construction of dominant repression and overexpression vectors

To construct a *GhKNL1* dominant repression vector of *Arabidopsis* (*GhKNL1-AtDR*), the coding sequence of *GhKNL1* in frame with the dominant repression sequence of *Arabidopsis EAR* (ethylene-responsive element binding factor-associated amphiphilic repression) was cloned into the pMD vector (Zhong *et al.*, 2008).

To construct a *GhKNL1* dominant repression vector of cotton (*GhKNL1-GhDR*), the coding sequence of *GhKNL1* was fused with the dominant repression sequence of cotton EAR, which shares high similarity with repression domain of *Arabidopsis* EAR, and cloned into pMD vector.

*GhKNL1*-overexpressing and complementation constructs were produced by inserting the coding sequence of *GhKNL1* downstream of CaMV (cauliflower mosaic virus) 35S and *4CL1* promoters in pMD and pCAMBIA1301 vectors, respectively. Primers are listed in Supplementary Table S1 available at *JXB* online. Constructs were introduced into cotton and *Arabidopsis*, respectively, by the transformation methods described previously (Li *et al.*, 2002; Gong *et al.*, 2012).

### Reverse-transcription PCR

Cotton total RNA was extracted from 20 DPA fibres and purified using a RNeasy Mini kit (Qiagen, Hilden, Germany). *Arabidopsis* total RNA was isolated from the basal inflorescence stems following the protocol of TRIzol (Invitrogen, USA). Real-time quantitative reverse-transcription PCR was performed using the fluorescent intercalating dye SYBR Green in a detection system (Opticon 2, MJ Research) by the method described previously (Li *et al.*, 2005). In brief, total RNA was converted into cDNA by reverse-transcription (RT) PCR which was used as template in real-time quantitative RT-PCR (qRT-PCR). Reactions were performed using Real-time PCR Master Mix (Toyobo, Shanghai, China) according to the manufacturer’s instructions. Cotton polyubiquitin (*GhUBI1*, accession number EU604080) and *AtActin2* (AT3G18780) were used as controls. Amplification of the target genes was monitored in every cycle by SYBR-Green fluorescence, and the relative quantity of the target gene expression level was determined by the comparative Ct (cycle threshold) method. To achieve optimal amplification, the annealing temperature for every primer combination was optimized and the efficiency of reverse transcription was verified by melting curve analysis and confirmed on agarose gel. For every real-time qRT-PCR reaction, each of the samples was derived from at least five plants of the same transgenic line. Data presented in the real-time qRT-PCR analysis are mean and standard deviation of three biological replicates of plant materials and three technical replicates in each biological sample using gene-specific primers (Supplementary Tables S3 and S4 available at *JXB* online).

### Microscopy

Tissues from wild-type and transgenic plants were harvested, fixed, and embedded in low-viscosity Spurr’s resin (SPI-PON 812). The embedded samples were cut into 3-μm-thick sections using a Leica microtome. The sections were stained with 0.25% (w/v) toluidine blue (Sigma) for 5min, rinsed briefly in distilled water, and observed under a light microscope.

For lignin staining, hand sections from 6-week-old inflorescence stems of *Arabidopsis* plants were stained in 1% (w/v) phloroglucinol-HCl and observed immediately under a light microscope.

For transmission electron microscopy (TEM), tissue embedding was carried out as described previously (Li *et al.*, 2011). Ten plants of each *GhKNL1* transgenic line grown in greenhouse were chosen for analysis. Measurement of cell-wall thickness in the wild type and four transgenic lines (*n*>40 cells for each individual line) was taken from micrographs of cells using ImageJ (http://rsb.info.nih.gov/ij/index.html) at standardized positions. For cotton fibres, the 1-μm cross-sections were sampled at 4 and 20 DPA and in mature fibre cells. For *Arabidopsis* stems, the 1-μm cross-sections were sampled from the bottom of 6-week-old inflorescence stems.

For seed coat mucilage analysis, *Arabidopsis* seeds were stained with 0.2% (w/v) aqueous ruthenium red solution and observed under a light microscope. Phenotypes of transgenic seeds were examined in the T1 generation and confirmed in generations T2–T4.

### *In vitro* culture of cotton ovules and TFU analysis

Cotton bolls at 1 DPA were soaked in 70% (v/v) aqueous alcohol to sterilize for 1–2min, followed by washing three times with sterile distilled water. The ovules picked from the sterilized bolls were placed in liquid BT medium supplemented with 0.5 μM gibberellic acid 3 and 5 μM indole acetic acid (Beasley and Ting, 1973). The ovules were cultured in dark at 30 °C for 20 days. Five ovules were placed in each Petri dish with liquid BT medium that was replaced by fresh medium once per week.

For total fibre unit (TFU) analysis, cultured ovules of both wild-type and transgenic plants were stained with 0.02% (w/v) toluidine blue (Sigma) for 30 seconds, washed with running water for 1min, and dried (Beasley *et al.*, 1974). Then ovules (10 for each measurement) were eluted with acetic acid/ethanol/water (10:95:5, v/v) for 2h and the extracted solution was assayed by measuring absorbance at 624nm using a spectrophotometer for further analysis. Each experiment was repeated three times.

## Results

### Isolation and characterization of cotton *KNL1*

By screening the fibre cDNA library, one full-length cDNA encoding a knotted-like homeobox protein (KNOX) was identified in cotton and designated as *GhKNL1* (accession number KC200250). Subsequently, its corresponding gene was isolated from cotton genome by PCR. This work further isolated 951bp of 5′-flanking sequence (including putative promoter fragment and 5′-untranslated region) of *GhKNL1* by genome-walking PCR. Thus, the nucleotide sequence of *GhKNL1* was 2248bp in length, including 951bp of 5′-flanking region, 903bp of coding sequence without intron, and 394bp of 3′-untranslated region. *GhKNL1* encoded a KNOX protein of 300 amino acids. Motif scan analysis identified that GhKNL1 consisted of four conserved domains: MEINOX domain, which could be divided into two subdomains (KNOX1 and KNOX2), a GSE domain, a ELK domain, and a homeodomain (Supplementary Fig. S1A available at *JXB* online).

GhKNL1 shared 91.0% identity with PoptrKNAT7 (POPTR_0001s08550; http://www.phytozome.net), 80.0% with *Arabidopsis* KNAT7 (At1g62990), and 71.0% with rice HOS66 (BAB55660.1) (Supplementary Fig. S1B available at *JXB* online). The phylogenetic relationship between cotton KNL1 and other KNOX proteins reported in *Arabidopsis* and rice is shown in Supplementary Fig. S1C available at *JXB* online. GhKNL1 had the highest homology with class II KNOXs PoptrKNAT7 and AtKNAT7. Based on the similarity of certain residues within the homeodomain and the phylogenetic relationship, the isolated GhKNL1 belonged to class II KNOX.

### Subcellular localization of GhKNL1

**Fig. 1.**
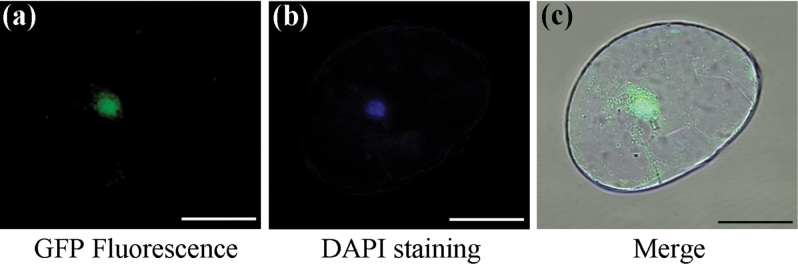
Subcellular localization of GhKNL1 in the cotton cell. Green fluorescence signals were localized to the cell nucleus of the *GhKNL1:GFP* transgenic cotton callus cell. (A) Confocal microsopy of GFP fluorescence. (B) Nuclear DAPI staining of the same cell in A. (C) A and B superimposed over the bright-field image. Bars=50 μm.

To determine the subcellular localization of GhKNL1, the *35S:GhKNL1:eGFP* vector was constructed and transferred into cotton by *Agrobacterium*-mediated transformation (Li *et al.*, 2002). After subculture for 3 months, stable transformed cotton callus cells were selected on a selective MS medium. The cells were stained with DAPI, and GFP fluorescence and DAPI staining in the cells were detected by confocal laser scanning microscopy. As shown in Fig. 1, both green GFP fluorescence and blue DAPI staining were clearly observed in cell nuclei of cotton cells, indicating that GhKNL1 was localized to the cell nucleus.

### *GhKNL1* was predominantly expressed in developing fibres

**Fig. 2.**
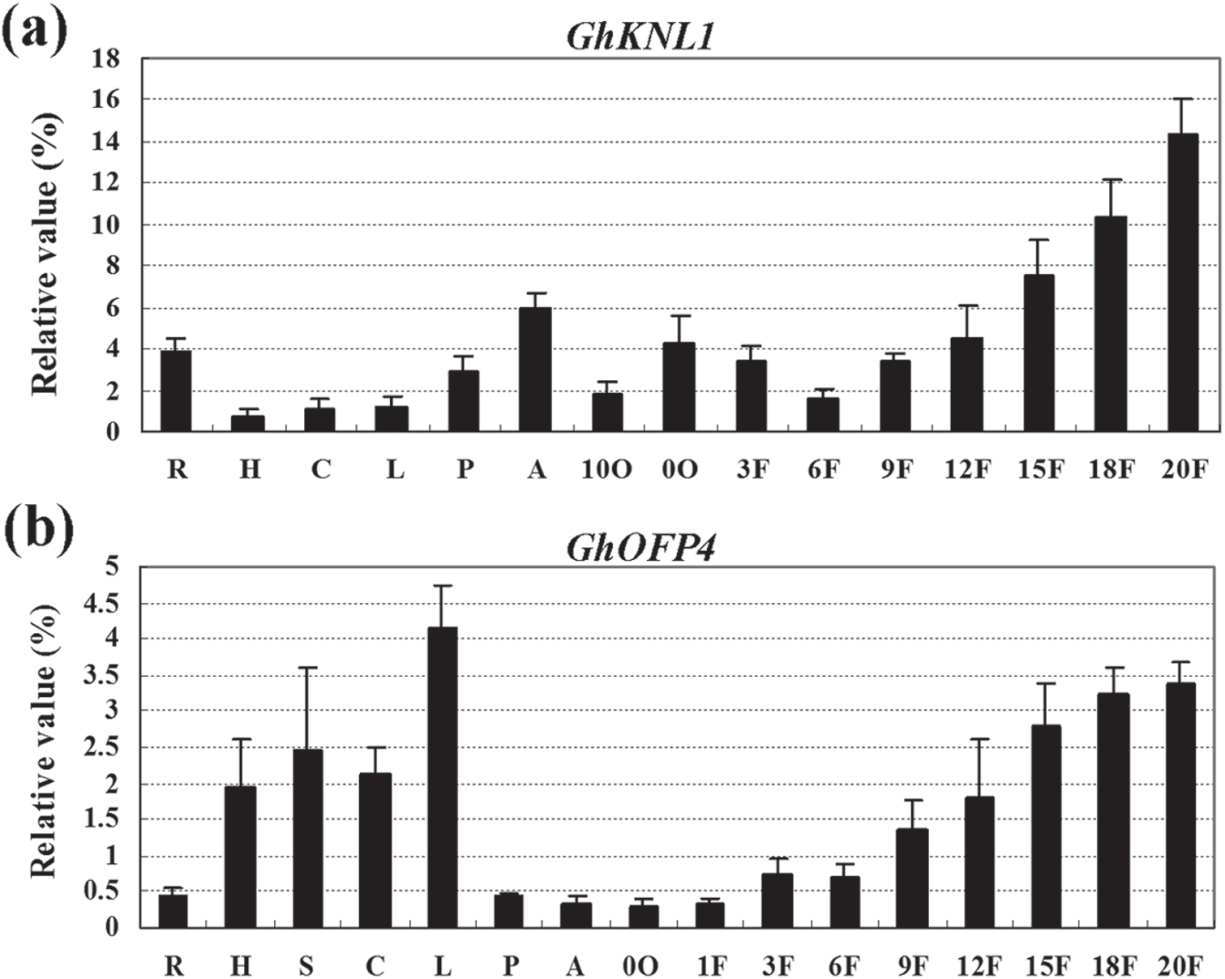
Real-time quantitative RT-PCR analysis of expression of *GhKNL1* (A) and *GhOFP4* (B) in different cotton tissues. Total RNA was isolated from tissues. Relative values of expression of *GhKNL1* and *GhOFP4* in cotton tissues are shown as percentage of *GhUBI1* expression activity; error bars represent standard deviation. Each analysis was reproduced for three independent experiments with three biological replicates of plant materials. DPA, days post anthesis; 0O, 0 DPA ovules; 10O, 10 DPA ovules; 12F, 12 DPA fibres; 15F, 15 DPA fibres; 18F, 18 DPA fibres; 1F, 1 DPA fibres; 20F, 20 DPA fibres; 3F, 3 DPA fibres; 6F, 6 DPA fibres; 9F, 9 DPA fibres; A, anthers; C, cotyledons; H, hypocotyls; L, leaves; P, petals; R, roots; S, stems.

The expression pattern of *GhKNL1* in different cotton tissues was analysed by RT-PCR. As shown in Fig. 2A, strong *GhKNL1* expression was found in developing fibres, whereas low-to-moderate expression was detected in roots, anthers, hypocotyls, cotyledons, leaves, petals, and ovules. *GhKNL1* transcripts were accumulated at a low level in early elongating fibres, but gradually reached a much higher level as fibres further developed to the SCW stage (15–20 DPA). These results suggested that expression of *GhKNL1* was regulated in developing fibres, especially at the SCW stage.

In addition, a cotton gene (*GhOFP4*) encoding OFP, which shares high similarity with AtOFP4, was isolated from cotton fibres. Real-time qRT-PCR analysis indicated that *GhOFP4* was strongly expressed in 15–20 DPA fibres and some vegetative organs/tissues, such as leaves, stems, and hypocotyls (Fig. 2B).

### Analysis of GhKNL1 transcriptional activation and its interaction with GhOFP4

**Fig. 3.**
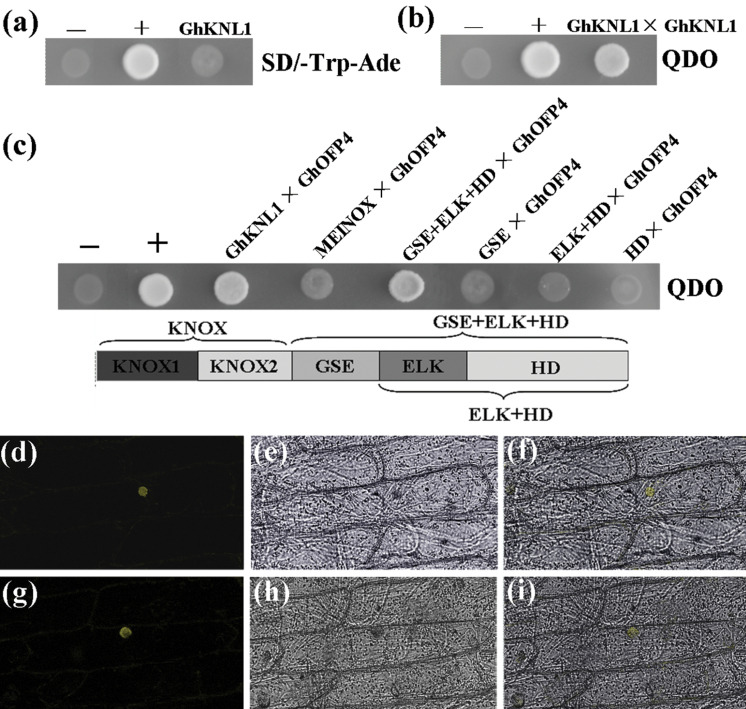
Analysis of transcriptional activation and interaction of GhKNL1 with GhOFP4. (A) Analysis of transactivation activity of GhKNL1 in yeast cells. Yeast transformants (AH109 containing PGADT7-KNL1) could not grow on SD/–Trp–Ade medium (SD minimal medium lacking Trp and Ade); +, positive control; –, negative control. (b) Yeast two-hybrid assay of GhKNL1–GhKNL1 interaction. The coding sequence of *GhKNL1* was cloned into the yeast two-hybrid vectors pGADT7 and pGBKT7, respectively, and introduced into yeast cells (see Methods). The interaction between two GhKNL1s was analysed by yeast mating. Transformants could grow on QDO nutritional selection medium (SD/–Trp–Leu–Ade–His medium); +, positive control; –, negative control. (c) Yeast two-hybrid assay of interaction between the full-length GhKNL1, segmented domains of GhKNL1, and GhOFP4 protein. Yeast transformants were assayed for growth on QDO nutritional selection medium. Zygotes grown on QDO medium, indicating that the two proteins could interact with each other. Otherwise, it means the proteins could not interact with each other. +, Positive control; –, negative control. (D–I) Bimolecular fluorescence complementation assay of GhKNL1–GhOFP4 interaction *in vivo*. GhKNL1:YFP-N and GhOFP4:YFP-C were transiently coexpressed in onion epidermal cells, using bZIP63 as positive control (see Methods). (D–F) BiFC visualization of bZIP63 dimerization in onion epidermal cells. (G–I) BiFC visualization of GhKNL1 interaction with GhOFP4 in onion epidermal cells. (D, G) YFP fluorescent images; (E, H) bright-field images of images D and G; (F and I) fluorescent images were merged with their bright-field images.

KNOX TFs contain N-terminal protein-interaction sites and a conserved C-terminal DNA-binding domain (the homeodomain), which can act as either a repressor or activator in regulatory pathways. Therefore this study employed a yeast two-hybrid system to analyse whether GhKNL1 had transcriptional activation activity. The pGBKT7-KNL1 fusion vector was transformed into strains AH109 and Y187. As shown in Fig. 3A, AH109 transformants did not grow on DDO medium, while Y187 transformants could not turn blue at the presence of 5-bromo-4-chloro-3-indolyl β-d-galactopyranoside (X-Gal), indicating that the reporter gene *LacZ* was not activated in yeast cells. These results suggested that GhKNL1 lacked transcriptional activation activity.

It has been reported that AtKNAT7 can interact with AtOFP4 to regulate SCW formation in *Arabidopsis* (Li *et al.*, 2011). As shown in Fig. 3B and C, zygotes from strain Y187 with pGBKT7-GhKNL1 mated with strain AH109 containing pGADT7-GhKNL1 or -GhOFP4 were able to grow well on QDO medium and turned blue at the presence of X-Gal. These results suggest GhKNL1 could combine into homodimers and interact with GhOFP4 in cells. Furthermore, six types of strain Y187 (containing the constructs pGBKT7-GhKNL1, -MEINOX, -GSE+ELK+homeodomain, -GSE, -ELK+homeodomain, and -homeodomain) were mated with strain AH109 (containing pGADT7-GhOFP4) and the zygotes were tested for growth on QDO medium. The results revealed that full-length GhKNL1 and the protein containing the GSE, ELK, and homeodomain domains could interact with GhOFP4 (Fig. 3C).

Additionally, further assays showed that GhOFP4 was also localized in the cell nucleus (Supplementary Fig. S2 available at *JXB* online). To further confirm the interaction between GhKNL1 and GhOFP4, bimolecular fluorescence complementation was employed. GhKNL1 was fused to the N-terminal half of eYFP, while GhOFP4 was fused to the C-terminal half of eYFP. Both proteins were transiently coexpressed in onion inner epidermal cells by particle bombardment-mediated transformation. The yellow fluorescence signals were observed in the cell nucleus after the onion epidermis was cultured on MS medium for 48h (Fig. 3G–I). The results demonstrated that GhKNL1 interacted with GhOFP4 *in vivo*.

### Dominant repression of *GhKNL1* affected fibre development of cotton

**Fig. 4.**
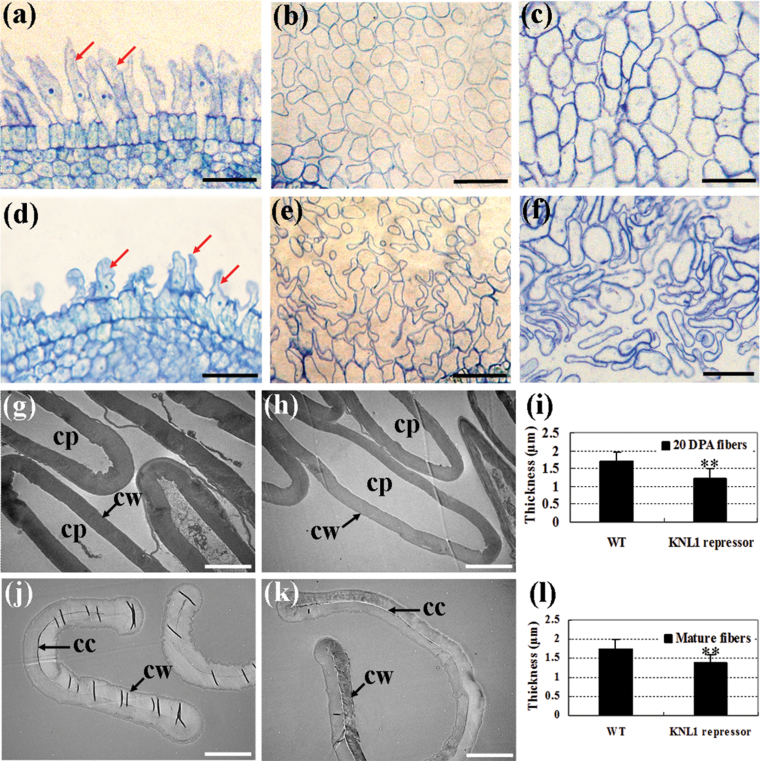
Comparison of fibre development between the dominant repression-*GhKNL1* transgenic cotton plants and the wild type. (A–F) Semi-thin sections (light micrographs) of developing fibres on ovules of transgenic plants and the wild type. (A) Sections of fibre initials in the middle zone of 1 DPA wild-type ovule surface; evenly arranged and spherically expanding fibre initials (indicated by arrows) on the ovule were observed. (B) Transections of 5 DPA fibres of the wild type; fibre cells displayed normal morphology. (C) Transections of 20 DPA fibres of the wild type; fibre cells displayed normal morphology. (D) Sections of fibre initials in the middle zone of 1 DPA transgenic ovule surface; fibre initials (arrows) on the ovule were much shorter and aberrant compared with the wild type. (E) Transections of 5 DPA transgenic fibres; aberrant, shrunken, and collapsed fibre cells were observed. (F) Transections of 20 DPA transgenic fibres; aberrant, shrunken, and collapsed fibre cells were observed. (G–L) Transmission electron microscope assay of SCW thickening in fibre cells of dominant repression-*GhKNL1* transgenic cotton plants and the wild type; 20 DPA (G–I) and mature fibre cells (J–L) were used for examining cell-wall thickness. (G, H) Transmission electron micrographs of transections of 20 DPA fibre cells; cell-wall thickness of 20 DPA fibres of *GhKNL1-GhDR* transgenic plants (H) was much less than that of the wild type (G). (I) Fibre cell-wall thickness; there was a significant difference (**P<0.01) in cell-wall thickness of 20 DPA fibres between transgenic T2 plants and the wild type. (J, K) Transmission electron micrographs of transections of mature fibre cells; the cell-wall thickness of mature fibres of *GhKNL1-GhDR* transgenic plants (K) was much less than that of the wild type (J). (L) Fibre cell-wall thickness; there was a very significant difference (**P value < 0.01) in cell-wall thickness of mature fibres between T2 transgenic plants and the wild type. Bars 10 μm (A, B, D, and E), 12 μm (C, F), and 5 μm (G, H, J, and K). cp, cytoplasm; cc, cell cavity; cw, cell wall; DPA, day post anthesis (this figure is available in colour at *JXB* online).

**Fig. 5.**
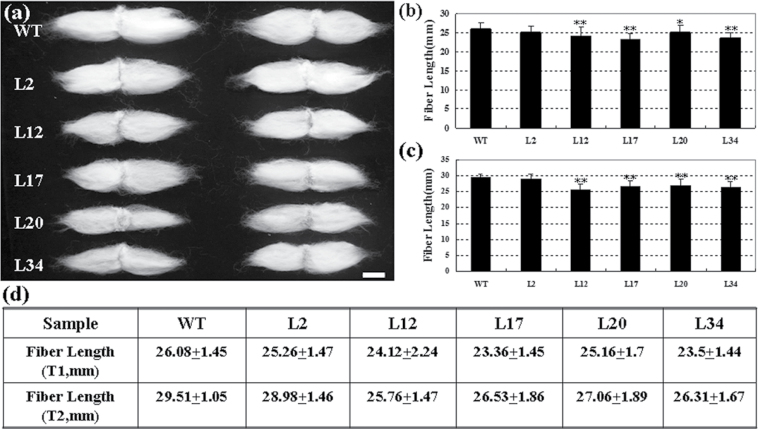
Analysis of mature fibre length in T1 and T2 transgenic cotton plants. Fibre length of transgenic lines was significantly shorter than that of the wild type. (A) Comparison of fibre length between T2 transgenic plants and the wild type. (B) Fibre length of T1 transgenic plants and the wild type. (C) Fibre length in T2 transgenic plants and the wild type. (D) Mean and standard deviation of fibre length are shown in T1 and T2 generations of the transgenic lines and wild type. Independent t-tests demonstrated that fibres of transgenic plants were significantly (*P<0.05) or very significantly (**P<0.01) shorter than those of the wild type. WT, wild type; L2, L12, L17, L20, and L34, transgenic lines (L2 with very low expression of *GhKNL1-GhDR* was used as negative control). Bar = 10 mm.

To investigate the role of *GhKNL1* in regulation of fibre development, chimeric repressor gene-silencing technology (CREST) was employed. CREST, belonging to the dominant repression approach, has been established on the basis of studies about the structure and function of EAR-type transcription repressors. Constructing chimeric proteins by linking the EAR-motifs to the C-end of TFs may genetically make them change into highly efficient negative regulons, which can be used to repress the expression of genes of interest specifically and efficiently. This approach is expected to block not only the functions of the TFs targeted for repression but also the functions of their homologues by competing with their binding to the same *cis*-elements or interacting proteins and has been successfully applied to facilitate the analysis of functionally redundant TFs involved in regulation of SCW biosynthesis in *Arabidopsis* (Hiratsu *et al.*, 2004; Zhong *et al.*, 2007, 2008). A dominant repression construct of *GhKNL1* was introduced into cotton via *Agrobacterium*-mediated transformation. Over 70 primary transgenic plants (T0) of 10 lines (L2, L12, L15, L17, L19, L20, L22, L31, L34, L57) were generated and shown to be positive by PCR (data not shown). Cosegregation analysis of generations T1 and T2 grown in field were performed by kanamycin selection. Genomic DNA and RNA was extracted and the expression of the segmented *GhKNL1* in frame with the specific dominant *EAR* repression sequence (*GhKNL1-GhDR(C)*, total 182bp) in transgenic fibres was analysed by PCR and real-time qRT-PCR. As shown in Supplementary Fig. S3 available at *JXB* online, transcripts were accumulated at different levels in 20 DPA fibres of the 10 transgenic lines (e.g. L34 showed the highest level, but L2 displayed a little expression), whereas no signal was detected in the wild type. Phenotypic and genetic analyses were performed in T2 transgenic cotton lines (including L2, L12, L17, L19, and L34). Over 40 plants from three lines (L12, L17, and L34) of transgenic cotton (generation T2) were chosen and fibres were sampled from the middle zone of the ovule surfaces. At 1–2 DPA, spherical fibre cells were evenly arranged on the surface of wild-type ovules (Fig. 4A). As they further developed, wild-type cotton plants displayed normal fibre cells (Fig. 4B, C). On the contrary, on surface of transgenic ovules, the fibre initials were much fewer and smaller. Compared with those of the wild type, a lot of fibre cells on the ovule surface were irregularly elongated and short (Fig. 4D). When they further developed, transgenic plants showed aberrant, shrunken, and collapsed fibre cells (Fig. 4E, F). TEM further demonstrated that these transgenic plants displayed reduced cell-wall thickness of fibres compared with the wild type. The cell-wall thickness of 20 DPA fibres in *GhKNL1-*dominant repression plants was 1.23±0.28 μm (Fig. 4G) and 1.71±0.25 μm in the wild type (*n*>40 cells for each individual line) (Fig. 4H). After fibres matured, transgenic fibre cell walls (1.38±0.20 μm) were still thinner than those of the wild type (1.73±0.28 μm) (Fig. 4J, K). Statistical analysis revealed a very significant difference in cell-wall thickness between transgenic and wild-type fibres (Fig. 4I, L). Additionally, mature fibre length was also determined. The fibre length of the T1 transgenic lines (23.36–25.16mm) was shorter than that of the wild type (26.08mm), except transgenic L2 (25.26mm), and this ‘short fibre’ phenotype still existed in the next generation (T2) of transgenic plants (Fig. 5).

**Fig. 6.**
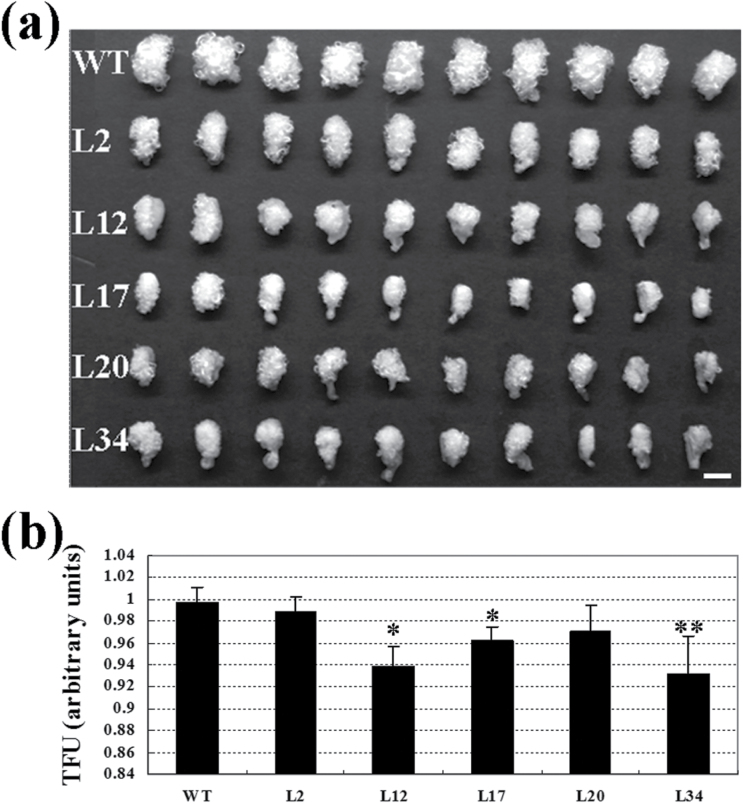
Total fibre unit (TFU) analysis of dominant repression-*GhKNL1* transgenic cotton plants. Cotton ovules at 1 DPA were cultured in liquid BT medium at 30 °C in darkness for 20 days. (A) Comparison of the phenotype of *in vitro* cultured ovules with fibres in the wild type (WT) and transgenic lines (L2, L12, L17, L20, and L34). (B) Fibre surface area measured by dye binding in TFU of *in vitro* cultured ovules. Independent t-tests demonstrated that there were significant differences (*P<0.05) or very significant differences (**P<0.01) in TFU between transgenic plants and the wild type. Bar = 0.5mm.

Total fibre unit was measured after 1 DPA cotton ovules were cultured at 30 °C in darkness for 20 days. Transgenic plants with normal phenotype at vegetative and reproductive stages were used for yield analysis. As shown in Fig. 6A, fibre growth in five *GhKNL1-GhDR* transgenic lines (L2, L12, L17, L19, and L34) was reduced to different degrees compared with the wild-type. TFU analysis indicated that fibre yield in transgenic plants was much lower (Fig. 6B). Most transgenic lines were unaffected in vegetative growth and flowering, but a few lines showed a leaf-curled phenotype. Some lines showed various degrees of suppression of fibre development, and bolls were smaller than those of the wild type (Supplementary Fig. S4 available at *JXB* online), but the vegetative growth and flower development of these transgenic lines were little affected. Collectively, the data suggested that dominant repression of *GhKNL1* affected cell elongation and SCW formation of cotton fibres.

### Dominant repression of *GhKNL1* affected expression of genes related to fibre development

**Fig. 7.**
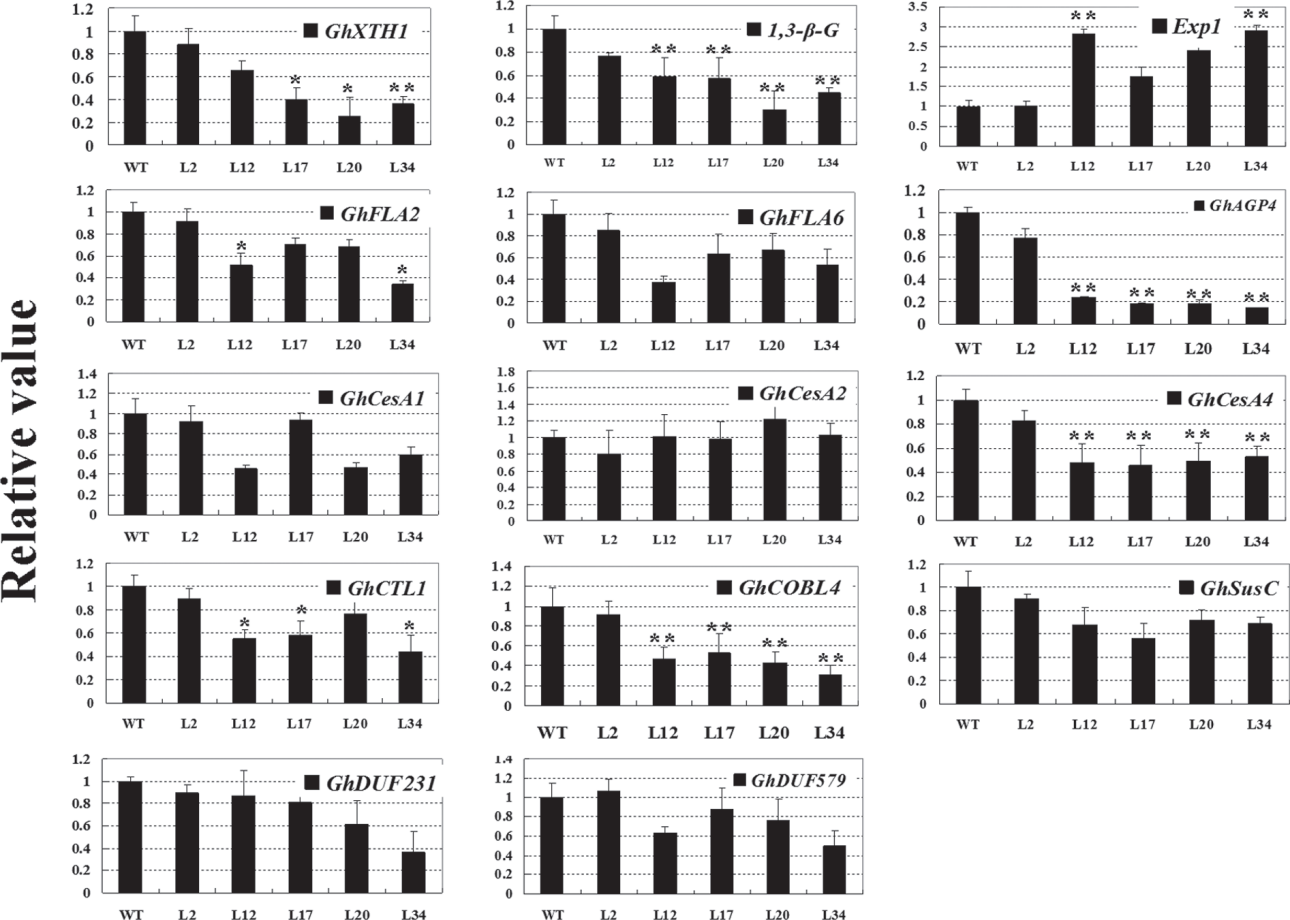
Real-time quantitative RT-PCR analysis of expression of genes related to fibre elongation and SCW synthesis in fibres of transgenic cotton plants. Total RNA was isolated from 20 DPA fibres of transgenic cotton plants (L2, L12, L17, L20, and L34) and the wild type (WT) and analysed by real-time quantitative RT-PCR; *GhUBI1* was used as a quantification control. Expression levels of fibre development-related genes in transgenic plants were normalized compared with WT. The examined genes were *XHT1* (xyloglucan endotransglycosylase/hydrolase 1), *1,3-β-G* (1,3-β-glucanase), *Exp1* (expansin1), *FLA2* (fasciclin-like arabinogalactan protein 2), *FLA6* (fasciclin-like arabinogalactan protein 6), *AGP4* (arabinogalactan protein 4), *CesA1* (cellulose synthase A1), *CesA2* (cellulose synthase A2), *CesA4* (cellulose synthase A4), *COBL4* (COBRA-like protein 4), *DUF579* (domain of unknown function family gene 579), *DUF231* (domain of unknown function family gene 231), *CTL1* (chitinase-like protein 1) and *SusC* (sucrose synthase C). Data are mean and standard deviation of three biological replicates of plant materials, and three technical replicates in each biological sample. Independent t-tests demonstrated that there were significant differences (*P<0.05) or very significant differences (**P<0.01) in gene expression between transgenic plants and the wild type.

The expression of several genes related to fibre elongation and SCW synthesis were further analysed in transgenic cotton fibres. As shown in Fig. 7, the expression of two fibre elongation-related genes, *XTH1* (xyloglucan endotransglycosylase/hydrolase 1) and *1,3-β-G* (1,3-β-glucanase), were suppressed to relatively low levels (30–80%) in the four transgenic lines with high *GhKNL1-GhDR* expression, compared with the wild type and the low *GhKNL1-GhDR*-expressing L2, consistent with the ‘short fibre’ phenotype of transgenic plants. On the other hand, transcripts of *Exp1* (expansin1) were remarkably upregulated, to at least 1.5–3-fold in transgenic fibres. As a result, transgenic plants displayed abnormal and disordered fibre elongation.

SCW synthesis of fibre cells approximately begins at 15 DPA (Wilkins and Arpat, 2005; Li *et al.*, 2010; Haigler *et al.*, 2012). To investigate whether GhKNL1 functions in regulating the process of SCW synthesis, several genes related to cellulose synthesis were analysed in transgenic cotton plants. As shown in Fig. 7, expression of *CesA1*, *CesA4*, *CTL1*, *COBL4* (COBRA-like protein 4), and *SusC* in transgenic fibres was decreased to 40–80% compared with the wild type and transgenic L2. Especially, *COBL4* transcripts in transgenic plants were declined to about one-third of that in the wild type. Similarly, expression of several cell-wall protein genes, such as *FLA2* (fasciclin-like arabinogalactan protein 2), *FLA6*, and *AGP4* (arabinogalactan protein 4), in transgenic fibres at the SCW-synthesis stage were much lower than in the wild type. However, there were little changes in transcription of *CesA2*, *DUF231* (domain of unknown function family gene 231), and *DUF579* in transgenic plants. These data suggested that GhKNL1 may regulate the process of SCW biosynthesis through modulating the expression of genes related to SCW biosynthesis during fibre development of cotton.

### Overexpression and dominant repression of *GhKNL1* in *Arabidopsis* affected SCW formation

**Fig. 8.**
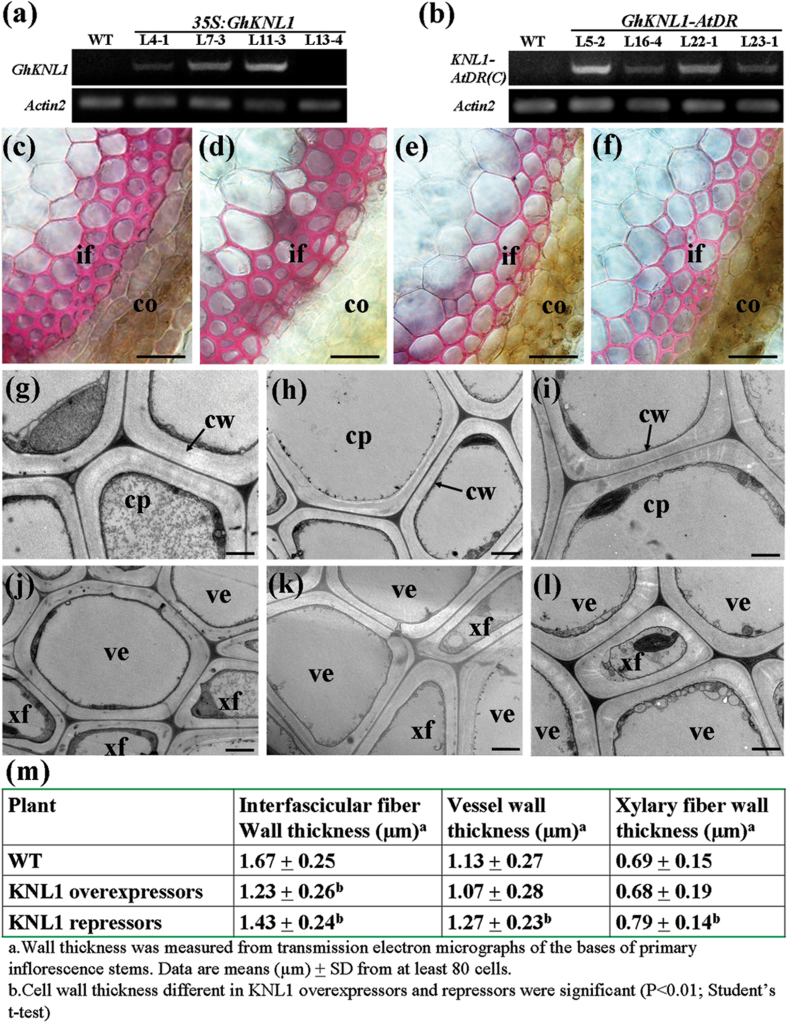
Assay of SCW thickening in fibres and vessels of *GhKNL1* transgenic *Arabidopsis* plants. The inflorescence stems of 6-week-old plants were used for examining SCWs of fibres and vessels. (A, B) Real-time quantitative RT-PCR analysis showing the presence of the *GhKNL1* overexpressor (*35S:GhKNL1*) and repressor (*GhKNL1-AtDR*) transcripts in the stems of four independent lines, respectively. Expression level of *Actin2* was used as a control. (C–F) Phloroglucinol-HCl staining of stem cross-sections of wild-type, *35S:GhKNL1*, and *GhKNL1-AtDR* transgenic seedlings: (C) wild type; (D) *35S:GhKNL1* transgenic line 13–4, negative control; (E) *35S:GhKNL1* transgenic line 11–3; (f) *GhKNL1-AtDR* transgenic line 5-2. (G–L) Transmission electron micrographs of stem transections of wild-type, *35S:GhKNL1*, and *GhKNL1-AtDR* transgenic plants, showing the thickened cell wall of interfascicular fibres (G–I) and vessels (J–L): (G, J) wild type; (H, K) *35S:GhKNL1* line 11–3; (I, L) *GhKNL1-AtDR* line 5-2. (M) SCW thickness of interfascicular fibres and vessels in stems of wild-type, *35S:GhKNL1* and *GhKNL1-AtDR* transgenic plants. Bar = 50 µm in (C–F) and 2 µm in (G–L). co, Cortex; if, interfascicular fibre; cp, cytoplasm; cw, cell wall; ve, vessel; xf, xylem fibre.

It has been indicated that cotton fibre SCW formation is similar to the process in *Arabidopsis* xylem (Betancur *et al.*, 2010). Therefore, *Arabidopsis*, as a model plant, was employed to investigate the role of *GhKNL1* in regulation of SCW formation. A *GhKNL1*-overexpressing construct (*35S:GhKNL1*) and a *GhKNL1-EAR* domain fusion with enhanced transcriptional repression (*GhKNL1-AtDR*) were introduced into *Arabidopsis*. Over 10 lines of *35S:GhKNL1* transgenic *Arabidopsis* were obtained, of which four lines (generation T2) with different *GhKNL1* expression levels were selected for further study (Fig. 8A). Likewise, histological examination of stems showed that a large portion of the *GhKNL1-AtDR* transgenic plants displayed significant reduction in the SCW thickening of interfascicular fibres and four lines (generation T2) with different *GhKNL1-AtDR* expression levels were selected for further study (Fig. 8B).

Histological staining showed that the wild type and transgenic line 13–4 (without *GhKNL1* expression, as a negative control) displayed normal morphology in basal stems (Fig. 8C, D). On the contrary, in both *35S:GhKNL1* and *GhKNL1-AtDR* transgenic plants there was a remarkable decrease in SCW thickness of interfascicular fibres (Fig. 8E, F). TEM further demonstrated that these transgenic plants displayed thinned cell walls in interfascicular fibres compared with the wild type (Fig. 8G–I). Cell-wall thickness of interfascicular fibres was 1.23±0.26 μm in *35S:GhKNL1* transgenic plants and 1.43±0.24 μm in *GhKNL1-AtDR* plants, compared with 1.67±0.25 μm in wild-type (*n*>40 cells for each individual line, total of four lines measured) (Fig. 8M). However, small changes in the SCWs of xylem fibres and vessels were observed in the transgenic lines compared with the wild type (Fig. 8J–M). Meanwhile, this work analysed overexpression of *GhKNL1* driven by the promoter of *4CL1* (*4CL1pro*), which can direct gene expression in cells with SCW thickening (Hauffe *et al.*, 1991). *4CL1pro:GhKNL1* transgenic *Arabidopsis* lines also showed more significant reduction in cell-wall thickness of the interfascicular fibres compared with the wild type (Supplementary Fig. S5A–C available at *JXB* online), similarly to *35S:GhKNL1* and *GhKNL1-AtDR* transgenic plants.

In addition, semiquantitative RT-PCR analysis revealed that the expression of lignin biosynthetic genes, such as *PAL1* (phenylalanine ammonia-lyase), *4CL1* (hydroxycinnamate CoA ligase), and *CcoAOMT1* (caffeoyl CoA O-methyltransferase), was remarkably decreased in *4CL1pro:GhKNL1-*overexpressing transgenic plants; however, expression of cellulose synthase genes, such as *CesA4*, *CesA7* and *CesA8*, and xylan biosynthetic genes, such as *FRAGILE FIBRE8* (*FRA8/IRX7*) and *IRREGULAR XYLEM 9* (*IRX9*), seemed to be little altered (Supplementary Fig. S5D available at *JXB* online). *GhKNL1-AtDR* seedlings also displayed similar expression profiling of SCW biosynthetic genes (Supplementary Fig. S5E available at *JXB* online). These results suggested that GhKNL1 may negatively regulate lignin biosynthesis in transgenic *Arabidopsis*.

### *GhKNL1* rescued the defective phenotype of *knat7* mutant

**Fig. 9.**
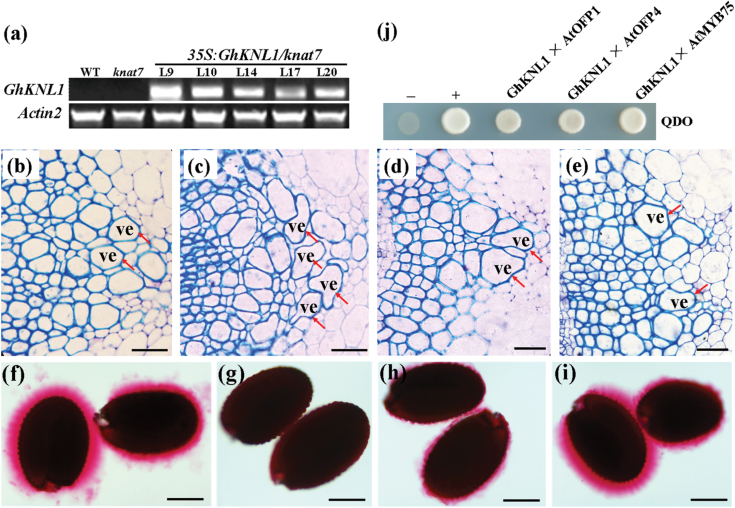
Complementation of *Arabidopsis knat7-3* mutant phenotype and analysis of GhKNL1 interaction with its partners. (A) RT-PCR analysis of expression of *35S:GhKNL1* in the *knat7-3* background and five independent transgenic lines, using the wild type and *knat7-3* mutant as negative controls. (B–E) Cross-sections of inflorescence stems; (F–I) mature seeds were stained with ruthenium red. (B, F) Wild type; (C, G) *knat7-3* mutant; (D, H) complementary *35S:GhKNL1*(*knat7*) transgenic line 14; (E, I) complementary *35S:GhKNL1*(*knat7*) transgenic line 9. Bar = 25 µm in (B–E) and 100 µm in (F– I). ve, vessel. Arrows in B indicate normal vessels in the wild type. Arrows in C–E indicate collapsed vessels in *knat7* mutant and complementary transgenic plants. (J) Analysis of GhKNL1 interaction with AtOFP1, AtOFP4, and AtMYB75, respectively; transformants could grow on QDO nutritional selection medium. +, Positive control; –, negative control.

A previous study showed that *knat7/irx11* mutant displayed obvious collapsed vessels and xylem fibres of basal stems and defective seed coat mucilage (Western *et al.*, 2000). To determine whether GhKNL1 could replace *Arabidopsis* KNAT7 function, the current work identified a T-DNA insertion loss-of-function mutant, SALK_110899 (*knat7-3*) and introduced *35S:GhKNL1* into the mutant (*35S:GhKNL1*(*knat7*)) for complementation. As shown in Fig. 9A, the expression levels in five representative *35S:GhKNL1*(*knat7*) lines were analysed by RT-PCR, and homozygous T3 progeny plants of lines L9 (high expression) and L14 (moderate expression) were selected for further study. Histological analysis revealed that wild-type plants had regular xylem in the basal inflorescence stems (Fig. 9B), whereas the *knat7* mutant displayed the irregular xylem phenotype (Fig. 9C). With expressing *GhKNL1* in the *knat7* mutant, the irregular xylem phenotype was abolished in the basal inflorescence stems of *35S:GhKNL1*(*knat7*) transgenic plants (Fig. 9D, E). Likewise, ruthenium red staining indicated that wild-type seeds had a thick mucilage layer (Fig. 9F), but *knat7* mutant seeds were devoid of a mucilage layer (Fig. 9G). This defect was partially or completely rescued in seeds of the complementary *35S:GhKNL1(knat7)* lines (Fig. 9H, I), which was consistent with the expression level of *GhKNL1* in transgenic plants. Additionally, yeast two-hybrid assay showed that cotton KNL1 could interact with AtOFP1, AtOFP4, and AtMYB75 (Fig. 9J), as did AtKNAT7 (Bhargava *et al.*, 2010, 2013; Li *et al.*, 2011). These results suggested that GhKNL1 may be capable of replacing AtKNAT7 function in transgenic *Arabidopsis*.

## Discussion

Plant KNOX proteins belong to a large conserved family which can be divided into three groups (class I, class II, and class III) based on phylogenetic clustering (Hake *et al.*, 2004; Magnani and Hake, 2008). Consistent with features of TFs, subcellular localization analysis showed that GhKNL1 was localized to the cell nucleus. It has been indicated that class I *KNOX* genes are characteristically expressed in the shoot apical meristem, whereas expression of class II *KNOX* genes is more widespread in plant tissues (Kerstetter *et al.*, 1994; Serikawa *et al.*, 1997). *AtKNAT3*, *AtKNAT4*, and *KNAT5* are expressed in different spatially restricted patterns along the longitudinal root axis and in lateral root primordials of *Arabidopsis* (Truernit *et al.*, 2006). Recently, a study indicated that class II *KNOX* genes *AtKNAT7* and *PoptrKNAT7* are strongly expressed in concert with SCW formation in *Arabidopsis* and *Populus*, respectively (Li *et al.*, 2012). Similarly, the current results showed that *GhKNL1* mRNAs are abundant in cotton fibres at the SCW-synthesis stage. These data suggested that GhKNL1 may participate in regulation of cotton fibre development, especially at the SCW stage.

Dimerization is important for the function of KNOX proteins. Several reports have showed that many homeodomain proteins require additional cofactors to bind with high affinity and specificity to their DNA sites (Vershon, 1996). Classical KNOXs possess four domains: MEINOX domain (including KNOX1 and KNOX2 subdomains), GSE domain, ELK domain, and the homeodomain. MEINOX and ELK domains are considered to act as a protein–protein interaction (Vollbrecht *et al.*, 1991). In addition, MEINOX and the homeodomain are necessary for homodimerization or heterodimerization, and the homeodomain, containing the WFIN sequence, is needed for binding of KNOX to its target sequence (Nagasaki *et al.*, 2001). Previous work suggested that BELL-KNAT proteins function as heterodimers, which might be mediated by AtOFP5 and is essential for normal development and cell specification in the *Arabidopsis* embryo sac (Pagnussat *et al.*, 2007). It is known that AtKNAT7 can interact with AtMYB75, forming a functional complex that contributes to regulating SCW deposition in *Arabidopsis* (Bhargava *et al.*, 2010, 2013). Moreover, AtKNAT7 interacts with AtOFP4 to enhance its repression transcription (Li *et al.*, 2011). In *Arabidopsis*, most members of the OFPs contain a predicted nuclear localization signal but lack a recognizable DNA-binding domain, and some of them have been found to interact with TALE proteins in a yeast two-hybrid screen, suggesting that AtOFP proteins are components of the TALE homeodomain protein network (Hackbusch *et al.*, 2005; Wang *et al.*, 2007). The current work demonstrated that GhKNL1 could interact with AtOFP4 and AtMYB75. The formation of this complex may be helpful to change the transcriptional activity of GhKNL1 for regulating downstream genes during SCW development. Similarly, conservative interactions of GhKNL1 also exist in cotton. These data revealed that GhKNL1 could form a homodimer and interact with GhOFP4 in cells, suggesting that GhKNL1 may form the heterodimer or/and homodimer to participate in signal transduction during fibre development of cotton.

The data presented in this study revealed that dominant repression of *GhKNL1* repressed fibre initiation and elongation to some extent. Transgenic cotton plants displayed less and small fibre initials and abnormal and short fibres with thinned cell walls compared with the wild type. Consistent with this phenotype, expression of genes related to fibre elongation and SCW synthesis were downregulated in transgenic fibres. The cellulose–hemicellulose network in the PCW plays an essential role in controlling extensibility of cell walls, and enzymes act on this network. For instance, XTH induces new xyloglucans and also causes hydrolytic cleavage of xyloglucan, and it is thought to be the enzyme that loosens cell walls for cell expansion and elongation (Michailidis *et al.*, 2009; Lee *et al.*, 2010). 1,3-β-glucanase hydrolyses 1,3-β-glucan into 3–5 glucose oligosaccharide units and glucose, participating in modification of cell walls during fibre development. Fibre elongation is initially achieved largely by cell-wall loosening and is finally terminated by increased wall rigidity and loss of higher turgor. Expansin is involved in coordination of turgor pressure, which dynamically promotes fibre elongation (Ruan *et al.*, 2001). In this study, the decreased expression of *XTH1* and *1,3-β-G*, and the increased expression of *Exp1* in transgenic cotton plants, may result in irregular loosening and collapsed fibre cells. Furthermore, expression of several genes related to cellulose biosynthesis and deposition, such as *GhCesA1*, *GhCesA4*, *GhCTL1*, and *GhCOBL4*, were decreased in transgenic fibres (Fig. 7). GhCesA-1 and GhCesA-2 proteins are believed to represent the catalytic subunit of cellulose synthase in cotton (Peng *et al.*, 2001). It is known that cotton *GhCTL1* and *GhCOBL4* are homologues of *Arabidopsis* SCW-related chitinase-like and cobra-like proteins (Zhang *et al.*, 2004). These proteins play vital roles in SCW cellulose synthesis and deposition (Somerville, 2006). In addition, a study reported that cotton *FLA2* and *FLA6* display low expression levels in fibres at early developmental stage and reach high levels in fibres at the SCW stage (Huang *et al.*, 2008). In the current study, dominant repression of *GhKNL1* also inhibited expression of genes (*FLA2*, *FLA6*, and *AGP4*) encoding arabinogalactan proteins. Up to now, the evidence has indicated that arabinogalactan proteins are related to cellulose deposition through changing the cytoskeleton in cotton fibre development. With depolymerization of actin filaments, distribution of both cellulose and arabinogalactan proteins are changed dramatically in pollen tubes of *Picea meyeri* (Chen *et al.*, 2007). The distribution and pattern of moving cellulose synthase (CesA) complexes are changed if microtubule polymerization is inhibited in transgenic *Arabidopsis* plants (Paredez *et al.*, 2006). Suppression of *GhAGP4* expression affects SCW synthesis through modulating cellulose deposition in cotton (Li *et al.*, 2010). Similarly, the current results suggested that dominant repression of cotton *KNL1* may affect cellulose biosynthesis in developing fibres through regulating expression of genes related to cell elongation and SCW biosynthesis of cotton fibres.

It has been suggested that cotton fibre SCW biosynthesis is similar to the process of xylem development in *Arabidopsis* (Betancur *et al.*, 2010). In this study, *GhKNL1* was introduced into *Arabidopsis* to analyse its potential function in SCW development of cotton fibres. Histological analysis showed that overexpression of *GhKNL1* in *Arabidopsis* resulted in a significant reduction in the interfascicular fibre wall thickening in transgenic plants. Expression of genes related to lignin biosynthesis was downregulated in *GhKNL1*-overexpressing transgenic plants. These results revealed that cotton *KNL1* may negatively regulate SCW formation in transgenic *Arabidopsis*. It seems a discrepancy that the *35S:GhKNL1* and *GhKNL1*-*AtDR* transgenic plants have the same phenotype of thinned cell walls of interfascicular fibres, being opposite to the phenotype of *knat7* mutant (Li *et al.*, 2012). The contradictory phenomenon also exists in *AtKNAT7-AtDR* and *4CL1pro:AtKNAT7* transgenic plants (Zhong *et al.*, 2008; Li *et al.*, 2012). The most probable explanation is that, as a transcriptional repressor, the EAR domain enhances AtKNAT7 repression function, generating a gain-of-function phenotype similar to that observed in AtKNAT7-overexpressing lines (Li *et al.*, 2011, 2012). Likewise, the same phenotype and similar expression levels of SCW synthesis-related genes exist in both *GhKNL1-AtDR* and *4CL1pro:GhKNL1* transgenic plants. Dominant repression of *GhKNL1* might enhance the inhibitory activity of GhKNL1, leading to a phenotype similar to that of *GhKNL1*-overexpressing *Arabidopsis*. In addition, yeast two-hybrid analysis showed that cotton KNL1 can interact with AtOFP1, AtOFP4, and AtMYB75. Complementation indicated that overexpression of *GhKNL1* in *knat7* mutant recovered the defective irregular xylem phenotype of stems and non-mucilage of seed coats to the wild-type phenotype to some extent. These data suggested that cotton *KNL1* gene may negatively regulate lignin synthesis and is functionally conserved in regulation of SCW formation in transgenic *Arabidopsis*. In contrast to the components of xylem in *Arabidopsis*, SCWs of mature fibres of cotton contain >90% cellulose (Haigler *et al.*, 2012). As a specialized cell type, cotton fibre may display its own distinct cell-wall property and characteristics. Given the data together, it is suggested that GhKNL1 may participate in regulation of fibre development through modulating expression of genes related to cell elongation and SCW biosynthesis of cotton fibres.

## Supplementary material

Supplementary data are available at *JXB* online.

Supplementary Table S1. Primer pairs used in mutant identification and the vectors construction for plant transformation.

Supplementary Table S2. Primer pairs used in the yeast two-hybrid and bimolecular fluorescence complementation constructs.

Supplementary Table S3. Primer pairs used in real-time quantitative RT-PCR analysis of cotton gene expression.

Supplementary Table S4. Primer pairs used in semiquantitative RT-PCR analysis of *Arabidopsis* gene expression.

Supplementary Fig. S1. Characterization of GhKNL1.

Supplementary Fig. S2. Subcellular localization of GhOFP4 protein in cotton cells.

Supplementary Fig. S3. Expression analysis of *GhKNL1-GhDR* in transgenic cotton plants.

Supplementary Fig. S4. Comparison of bolls in *GhKNL1* transgenic lines and the wild type.

Supplementary Fig. S5. Histology and expression of genes related to SCW biosynthesis in *GhKNL1* transgenic *Arabidopsis* plants.

Supplementary Data
