# Supplementary material for: Cotton KNL1, encoding a class II KNOX transcription factor, is involved in regulation of fibre development
Source: J Exp Bot. 2014 May 15;65(15):4133–47. doi: 10.1093/jxb/eru182 (PMC4112624; doi:10.1093/jxb/eru182)
Supplement: Supplementary Data [file supp_65_15_4133__index.html]

Cotton KNL1, encoding a class II KNOX transcription factor, is involved in regulation of fibre development — Cotton KNL1, encoding a class II KNOX transcription factor, is involved in regulation of fibre development — Supplementary Data 

# Cotton *KNL1*, encoding a class II KNOX transcription factor, is involved in regulation of fibre development

## Supplementary Data

Data files

**Files in this Data Supplement:**

- Supplementary Data - Supplementary Data
