# Supplementary material for: Cotton KNL1, encoding a class II KNOX transcription factor, is involved in regulation of fibre development
Source: J Exp Bot. 2014 May 15;65(15):4133–47. doi: 10.1093/jxb/eru182 (PMC4112624; doi:10.1093/jxb/eru182)
Supplement: Supplementary Data [file supp_eru182_jexbot115717_file001.pdf]

Supporting Information Tables S1– S4 and Figs S1 – S5

Table S1. Primer pairs used in mutant identification and the vectors construction for plant transformation.

Gene name	Primer sequence
AtKNAT7LP	5’-TTGCCACCAATTTTTCAAGAC-3’ (F)
AtKNAT7RP	5’-GCTTCAAAGAACAGCTGCAAC-3’ (R)
AtKNAT7RT	5’-GAGACAGGATTGCAGTTGAAGC-3’ (F)
	5’-TAACAACCAGTAATCACGCAAC-3’ (R)
GhKNL1-OE	5’-CTTGGATCCATGCAAGAACCAGGGTTAG-3’ (F)
	5’-CTTGTCGACCTACCGTTTTCGCTTCGAC-3’ (R)
GhKNL1-GFP	5’-CTTGGATCCATGCAAGAACCAGGGTTAG-3’ (F)
	5’-CTTGTCGACCCGTTTTCGCTTCGACTTTA-3’ (R)
GhOFP4-GFP	5’-CTTGGATCCATGGGTAATTCCAGGTTCAAG-3’ (F)
	5’-CTTTCTAGATCGGACATCGATCAAGTCGAA-3’ (R)
GhKNL1RT	5’-GCCAAACTTGTGGAGGAAACAG-3’ (F)
	5’-GAGACATACATCACAATAATGAG-3’ (R)
GhKNL1-AtDR	5’-CTTGGATCCATGCAAGAACCAGGGTTAG-3’ (F)
	5’-CTTGTCGACTTAAGCGAAACCCAAACGGAGTTCTAGATCCAG ATCCAGCCGTTTTCGCTTCGACTTTAAAG-3’ (R)
GhKNL1-AtDR (C)	5’-GCCAAACTTGTGGAGGAAACAG-3’ (F)
	5’-ACGGAGTTCTAGATCCAGATC-3’ (R)
GhKNL1-GhDR	5’-CTTGGATCCATGCAAGAACCAGGGTTAG-3’ (F)
	5’-CTTGTCGACTCAGCTCTGGGCGTTTAAACCAAGCCGGAGTTC CAGATCTATGTTGTTGTCCCGTTTTCGCTTCGACTTTAAAG-3’ (R)
GhKNL1-GhDR (C)	5’-GCCAAACTTGTGGAGGAAACAG-3’ (F)
	5’-ACCAAGCCGGAGTTCCAGATC-3’ (R)

F, forward; R, reverse.

Table S2. Primer pairs used in the yeast two-hybrid and BiFC constructs.

Gene name	Primer sequence
GhKNL1	5'-CTTGAATTCATGCAAGAACCAGGGTTAG-3' (F)
	5'-CTTCTGCAGCTACCGTTTTCGCTTCGAC-3' (R)
GhKNL1-MEINOX	5'-CTTGAATTCATGCAAGAACCAGGGTTAG-3' (F)
	5'-CTTCTGCAGTCCAGTAAGAGCTTGCAAGTT-3' (R)
GhKNL1-GSE+ELK+HD	5'-CTTGAATTCGTCACATTGGGTGAAGGAAC-3' (F)
	5'-CTTCTGCAGCTACCGTTTTCGCTTCGAC-3' (R)
GhKNL1-GSE	5'-CTTGAATTCGTCACATTGGGTGAAGGAAC-3' (F)
	5'-CTTCTGCAGCTGGCGAACCCTCTCCATT-3' (R)
GhKNL1-ELK+HD	5'-CTTGAATTCGAGCTCAAGATTGAATTG-3' (F)
	5'-CTTCTGCAGCTACCGTTTTCGCTTCGAC-3' (R)
GhKNL1-HD	5'-CTTGAATTCTTACGGAAAAGAAGGGCTGG-3' (F)
	5'-CTTCTGCAGCTACCGTTTTCGCTTCGAC-3' (R)
GhOFP4	5'-GGGCATATGATGGGTAATTCCAGGTTCAAG-3' (F)
	5'-CTTGGATCCTCATCGGACATCGATCAAGTC-3' (R)
AtOFP1	5'- GGGCATATGATGGGTAATAACTATCGGTT-3' (F)
	5'- CTTGAGCTCTTATTTGGAATGGGGTGGTG-3' (R)
AtOFP4	5'- GGGCATATGATGAGGAACTATAAGTTAAG-3' (F)
	5'- CTTGAGCTCCTACTTCGATGCAAATGTAG-3' (R)
AtMYB75	5'-CTTCATATGATGGAGGGTTCGTCCAAAGG-3' (F)
	5'- GCCGGATCCCTAATCAAATTTCACAGTCT-3' (R)
YNE-GhKNL1	5'- CTTGGATCCATGCAAGAACCAGGGTTAG-3' (F)
	5'- CTTCTCGAGCCGTTTTCGCTTCGACTTT -3' (R)
YCE-GhOFP4	5'-CTTGGATCCATGGGTAATTCCAGGTTCAAG-3' (F)
	5'-CTTCTCGAGGAATAAACGTCTGCATCCAC-3' (R)

Note: GhOFP4 was identified in our lab, and expression level of the gene is gradually increased during fiber development. F, forward; R, reverse.

Table S3. Primer pairs used in quantitative RT-PCR analysis of cotton gene expression.

Gene name	Accession no.	Primer sequence
GhXTH1	HM749062.1	5'-GTGCCAGGCCCAGCAA-3' (F)
		5'-GGCATTAAGGGCTTGATAAGTAGATC-3' (R)
Gh1,3-ß-G	CAA92278.1	5'-GGTTTGAACGGCAACAATCT-3' (F)
		5'-TTGATCTTTTGCGAGGCTTT-3' (R)
GhExp1	AY189969	5'-ATGCTTACCTTAACGGCCAAAGCC-3'(F)
		5'-AGTTTGTCCGAATTGCCAACCAGC-3' (R)
GhFLA2	EF470297	5'-GAAAGTTTCCAGCGCTGTTCAC-3' (F)
		5'-CAACATGAACATAAATTAGACTCG-3' (R)
GhFLA6	ABV27477	5'-GTGGGAAGTTTCCGCTTAATGT-3' (F)
		5'-TCACAATCACAAATTACAAACAG-3' (R)
GhAGP4	EF470295	5'-ACCGGGAATGTCTAGGAAGG-3' (F)
		5'-CCATAGGCAATAAGTGGAGG-3' (R)
GhCesA1	U58283	5'-TGGACTACCCGGTGGATAAGGT-3' (F)
		5'-CTTTCTTGCAAAGTCGGCTGTT-3' (R)
GhCesA2	AAF72619.1	5'-TCTGATAATACTGAACATGGTCGGAGT-3' (F)
		5'-GAAATTAAATTGAACCAACAAAATCATAGG-3' (R)
GhCesA4	U58284	5'-GCAGCAGACGATACAGAATTCG-3' (F)
		5'-CGTTGTTGATTGCGTCTGAAAC-3' (R)
GhCTL1	AY291285	5'-CCGACCAAGAACGACACGTT-3' (F)
		5'-ACCTCGCCCACAAACTTGAT-3' (R)
GhCOBL4	CO496020	5'-GGCATGAAGTTCTACAATGAC-3' (F)
		5'-AATCACCAAACAGTGACCAAC-3' (R)
GhSusC	JN248435.1	5'-CAATGGGACCAAACCCAGAGTTC-3' (F)
		5'-AGCAAAAGGCTGCTTGGAAAC-3' (R)
GhDUF579		5'-CAGAATGCAACCGATCTTCAC-3' (F)
		5'-ACAACGACTACGAAACTGAAG-3' (R)
GhDUF231		5'-CAGCAGTTGCCTACCAAATAG-3' (F)
		5'-ACCACACTCATCATCTCCTTG-3' (R)
GhKNL1DR		5'-GCCAAACTTGTGGAGGAAACAG-3' (F)
		5'-GGAGTTCCAGATCTATGTTGTT -3' (R)
GhUBI1	EU604080	5'-CTGAATCTTCGCTTTCACGTTATC-3' (F)
		5'-GGGATGCAAATCTTCGTGAAAAC-3' (R)

Note: GhDUF579 and GhDUF231 were identified in our lab, and both genes are expressed in late developing fibers at secondary cell wall. F, forward; R, reverse.

Table S4. Primer pairs used in semi-quantitative RT-PCR analysis of *Arabidopsis* gene expression.

Gene name	Accession no.	Primer sequence
AtPAL1	At2g37040	5'-TAGATTCGTGAGGGAAGAGCT-3' (F)<br>5'-CCACTTCACAGACAATCATTTGG-3' (R)
AtCCoAoMT1	At4g34050	5'-TCGTTGATGCTGACAAAGACA-3'(F)<br>5'-ACTGATCCGACGGCAGATAG-3' (R)
At4CL1	At1g51680	5'-GGTTACCTCAACAATCCGGCA-3'(F)<br>5'-CAAATGCAACAGGAACTTCAC-3' (R)
AtCesA4	At5g44030	5'-CTCAACATGGTCGGTGTTGTTG-3' (F)<br>5'-TCGACGCCACATTGCTTCAGT-3' (R)
AtCesA7	At5g17420	5'-TTGTTGCAGGCATCTCAGATG-3' (F)<br>5'-GCAGTTGATGCCACACTTGGA-3' (R)
AtCesA8	At4g18780	5'-TGAGCTTTACATTGTCAAATG-3' (F)<br>5'-GCAATCGATCAAAAGACAGTT-3' (R)
AtIRX7/FRA8	At2g28110	5'-GTCCAAACCATAGATCCGTTCT-3' (F)<br>5'-GATCTGTGAATCTCCTCTCTG-3' (R)
AtIRX9	At2g37090	5'-TGAGGCACATTGAACATCACAA-3' (F)<br>5'-ATCTTGTGCCGGAAGTCCCTT-3' (R)
AtACTIN2	At3g18780	5'-GAAATCACAGCACTTGCACC-3' (F)<br>5'-AAGCCTTTGATCTTGAGAGC-3' (R)

F, forward; R, reverse.

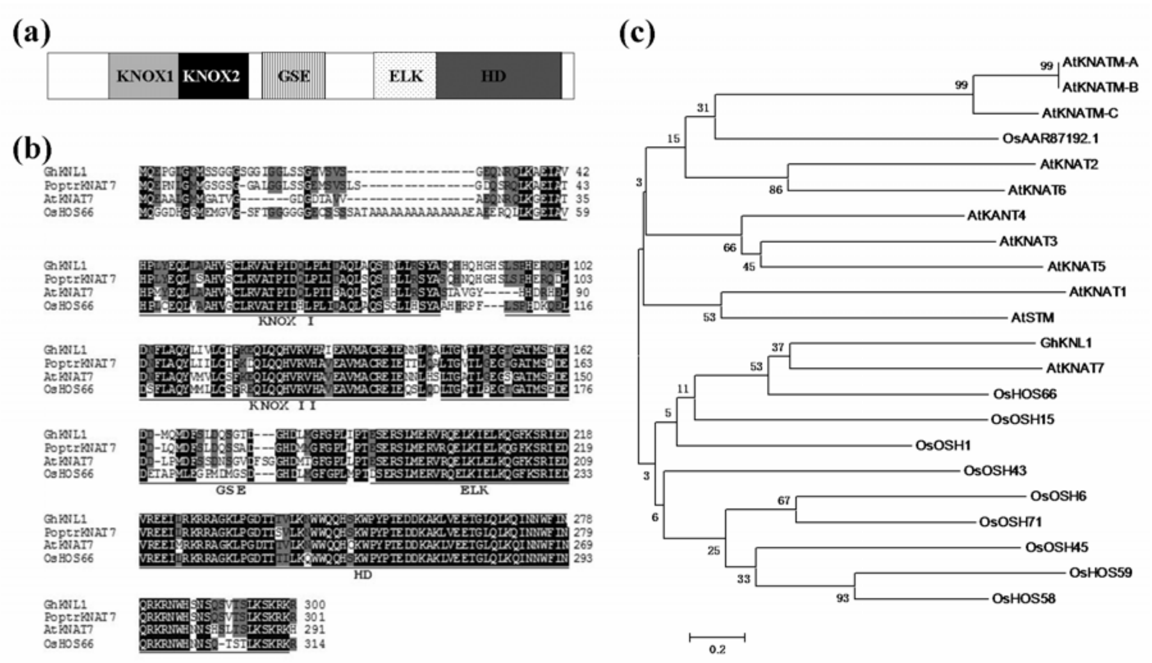

Figure S1. Characterization of GhKNL1. (a) Schematic diagram of the domain structure of the deduced cotton KNL1 protein. GhKNL1 protein is divided into four regions. The regions indicated are KNOX1 (light-gray), KNOX2 (black), GSE domain (white with strips), ELK domain (white with spots) and homeodomain (HD, dark-gray). (**b**) Comparison of the predicted amino acid sequence of cotton KNL1 with some known KNOXs in *Populus*, *Arabidopsis* and rice. The alignment was generated by ClustalW program. The same amino acids are highlighted in black, conserved and similar residues are indicated in gray. KNOX1, KNOX2, GSE, ELK, and HD regions are underlined. **(c)** Neighbor-joining tree was generated by MEGA5 from 1000 bootstrap replicates. Sequence data from this article can be found in the EMBL/GenBank data libraries under accession numbers as follows: PoptrKNAT7 (POPTR_0001s08550; http://www.phytozome.net), AtSTM (At1g62360), AtKNAT1/BP (At4g08150), AtKNAT2 (At1g70510), AtKNAT3 (At5g25220), AtKNAT4 (At5g11060), AtKNAT5 (At4g32040), AtKNAT6 (At1g23380), AtKNAT7 (At1g62990), AtKNATM (At1g146760), OsAAR87192.1, OsHOS66 (BAB55660.1), OsOSH1 (P46609.2), OsOSH6 (BAA79224.1), OsOSH15 (BAA31688.1), OsOSH43 (BAA79225.1), OsOSH45 (BAA08552.1), OsOSH71 (BAA79226.1), OsHOS58 (Q0E3C3.2), OsHOS59 (BAB55659.1), and OsHOS66 (BAB55660.1).

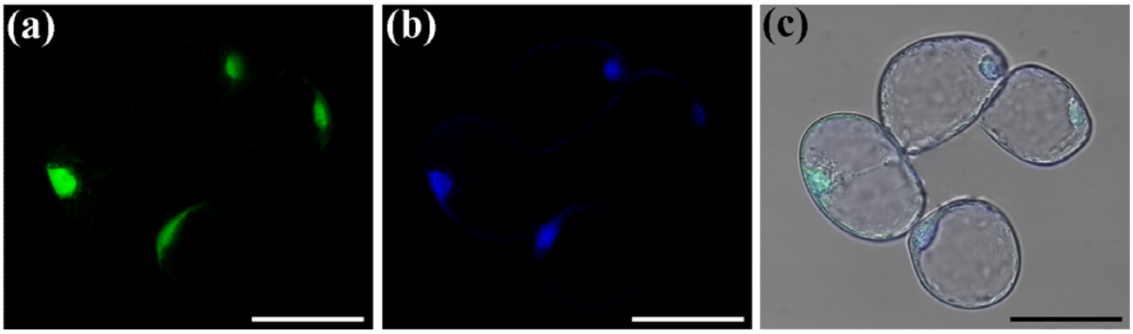

Figure S2. Subcellular localization of GhOFP4 protein in cotton cells. Green fluorescence signals were localized in the cell nucleus of the *GhKNL1:GFP* transgenic cotton callus cells. (**a**) Confocal microsopy image of GFP fluorescence; (**b**) Nuclear DAPI staining of the same cotton cells in image A; (**c**) Image A and B superimposed over the bright field image are shown. *Bar* = 50 μm.

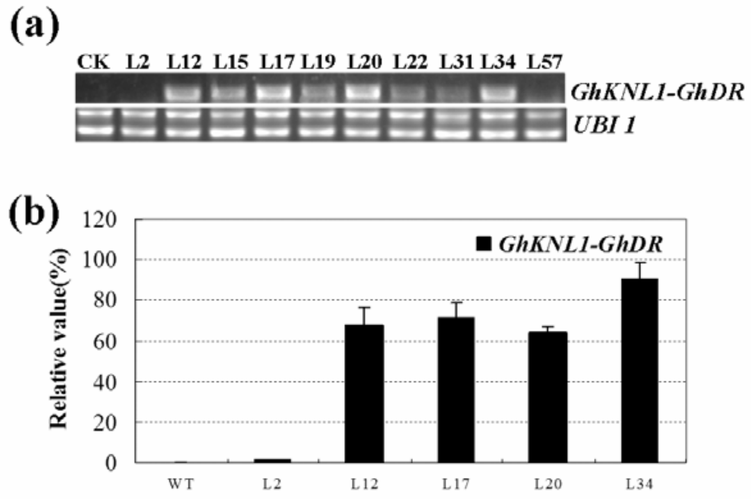

Figure S3. Expression analysis of *GhKNL1-GhDR* in transgenic cotton plants. (a, b) Analysis of the transcripts of *GhKNL1DR* sequence in fibers of the transgenic plants and wild type, using *GhUBI1* as a quantification control. *GhKNL1DR* transcripts were detected in the transgenic 20 DPA fibers, but not in wild type. WT, wild type; L2 – L34, transgenic lines. Error bars represent standard deviation.

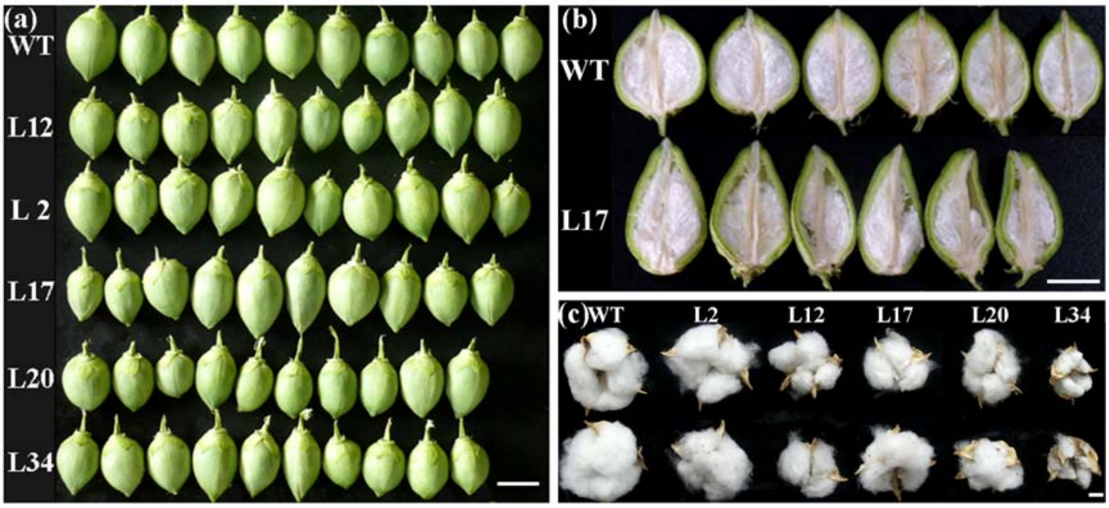

Figure S4. Comparison of the bolls between *GhKNL1* transgenic lines and wild type. **(a)** 20 DPA bolls of transgenic plants were smaller than those of wild type. **(b)** Ovule-abortive phenotype was observed in 20 DPA bolls of the transgenic plants. **(c)** The mature bolls of transgenic plants (T2) were smaller than those of wild type. Bars = 2 cm in **(a, b)** and 1 cm in **(c)**.

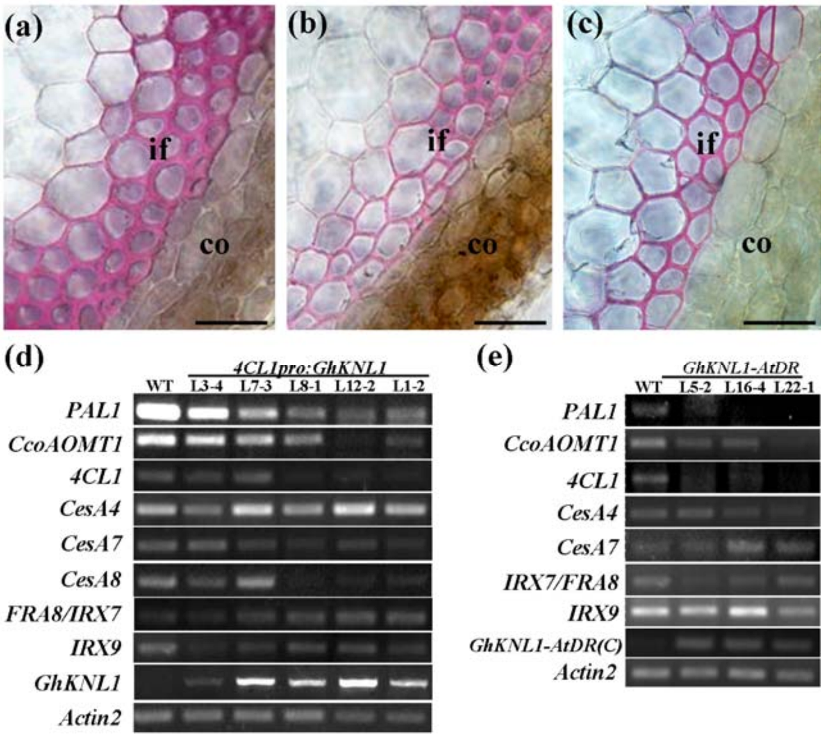

Figure S5. Analyses of histology and expression of the genes related to secondary wall biosynthesis in *GhKNL1* transgenic *Arabidopsis* plants. (a–c) Phloroglucinol-HCl staining of the stem cross-sections in wild type and *4CL1pro:KNL1* transgenic seedlings. **(a)** Wild type; **(b)** Transgenic line 7-3; **(c)** Transgenic line 8-1. **(d, e)** RT-PCR analysis of expression of the genes related to secondary wall biosynthesis in 6-week-old flower inflorescence stems of *4CL1pro:KNL1* transgenic plants (lines L3-4, L7-3, L8-1, L12-2 and L1-2) **(d)** and *KNL1-AtDR* transgenic lines (L5-2, L16-4, and L22-1) **(e)**, compared with the wild type (WT), using *Actin2* as standard control. The genes includes *PAL1* (phenylalanine ammonia lyase 1), *4CL1* (4-coumarate CoA ligase 1), *CCoAoMT1* (caffeoyl CoA 3-*O*-methyltransferase 1), *CesA4* (cellulose synthase A 4), *CesA7* (cellulose synthase A 7), *CesA8* (cellulose synthase A8), *FRA8/IRX7* (FRAGILE FIBER8), and *IRX9* (IRREGULAR XYLEM 9). Scale Bars = 50 µm in **(a–c)**. co, cortex; if, interfascicular fiber.
